# Effect of postnatal low-dose exposure to environmental chemicals on the gut microbiome in a rodent model

**DOI:** 10.1186/s40168-016-0173-2

**Published:** 2016-06-14

**Authors:** Jianzhong Hu, Vincent Raikhel, Kalpana Gopalakrishnan, Heriberto Fernandez-Hernandez, Luca Lambertini, Fabiana Manservisi, Laura Falcioni, Luciano Bua, Fiorella Belpoggi, Susan L.Teitelbaum, Jia Chen

**Affiliations:** Department of Genetics and Genomic Sciences, Icahn School of Medicine at Mount Sinai, New York, NY USA; Department of Preventive Medicine, Icahn School of Medicine at Mount Sinai, New York, NY USA; Department of Pediatrics, Icahn School of Medicine at Mount Sinai, New York, NY USA; Department of Oncological Sciences, Icahn School of Medicine at Mount Sinai, New York, NY USA; Cesare Maltoni Cancer Research Centre, Ramazzini Institute, Bentivoglio, Bologna, Italy

**Keywords:** Phthalate, Paraben, Triclosan, Microbiota

## Abstract

**Background:**

This proof-of-principle study examines whether postnatal, low-dose exposure to environmental chemicals modifies the composition of gut microbiome. Three chemicals that are widely used in personal care products—diethyl phthalate (DEP), methylparaben (MPB), triclosan (TCS)—and their mixture (MIX) were administered at doses comparable to human exposure to Sprague-Dawley rats from birth through adulthood. Fecal samples were collected at two time points: postnatal day (PND) 62 (adolescence) and PND 181 (adulthood). The gut microbiome was profiled by 16S ribosomal RNA gene sequencing, taxonomically assigned and assessed for diversity.

**Results:**

Metagenomic profiling revealed that the low-dose chemical exposure resulted in significant changes in the overall bacterial composition, but in adolescent rats only. Specifically, the individual taxon relative abundance for *Bacteroidetes* (*Prevotella*) was increased while the relative abundance of *Firmicutes (Bacilli)* was reduced in all treated rats compared to controls. Increased abundance was observed for *Elusimicrobia* in DEP and MPB groups, *Betaproteobacteria* in MPB and MIX groups, and *Deltaproteobacteria* in TCS group. Surprisingly, these differences diminished by adulthood (PND 181) despite continuous exposure, suggesting that exposure to the environmental chemicals produced a more profound effect on the gut microbiome in adolescents. We also observed a small but consistent reduction in the bodyweight of exposed rats in adolescence, especially with DEP and MPB treatment (*p* < 0.05), which is consistent with our findings of a reduced *Firmicutes/Bacteroidetes* ratio at PND 62 in exposed rats.

**Conclusions:**

This study provides initial evidence that postnatal exposure to commonly used environmental chemicals at doses comparable to human exposure is capable of modifying the gut microbiota in adolescent rats; whether these changes lead to downstream health effects requires further investigation.

**Electronic supplementary material:**

The online version of this article (doi:10.1186/s40168-016-0173-2) contains supplementary material, which is available to authorized users.

## Background

Microbes that live on and inside the human body (microbiota) comprise about 100 trillion microbial cells [1–3]; the ratio of human to bacterial cells in the body is estimated to be approximately 1 to 1 [4]. Commensal bacteria provide a wide range of metabolic functions that the human body lacks. They facilitate diverse processes such as digestion of the nutrients and production of short-chain fatty acids and offer protection against pathogen colonization through competition for nutrients, secretion of antimicrobial substances, and microniche exclusion [5]. Commensal bacteria in the gut also promote angiogenesis and development of the intestinal epithelium; these bacteria have been shown to be essential for the normal development and function of the immune system [5]. However, the mechanistic relationship between microbiota diversity and biological function under different settings of host genetics or environmental factors remains obscure. Accumulating evidence suggests that the identity and relative abundance of many taxa in microbial communities are associated with environmental factors including diet and antibiotics [6–10]. How exposure to exogenous chemicals may influence the microbiome remains to be studied.

Human exposure to environmental chemicals is ubiquitous, and one major exposure source is through the use of personal care products. The most recent NHANES survey of environmental chemical exposure demonstrates measurable concentrations of diethyl phthalate (DEP), methylparaben (MPB), and triclosan (TCS) in the vast majority of the US population [11]. These three chemicals are frequently added to personal care products; DEP is used to stabilize fragrances and increase plastic flexibility, while MPB and TCS are commonly added as preservatives and microbicides [11]. Exposure to these chemicals has been linked to various health effects including obesity and other metabolic diseases [12–15] as well as breast cancer [16]. However, the underlying mechanism of these associations has not been clearly elucidated. Although the term “endocrine disruptors” has often been used to describe these chemicals, the evidence demonstrating such properties is conflicting and far from conclusive.

Emerging evidence suggest that the gut microbiome plays a critical role in human metabolism [17]. Importantly, the core microbiome is believed to be established over the first few years of life in humans, and its composition is susceptible to exogenous factors including diets and antibiotics [18, 19]. Early exposures are important, as denoted in the Developmental Origins of Health and Disease (DOHaD) paradigm. In particular, the adolescent period represents a narrow but profoundly critical window of susceptibility to myriad environmental exposures and conditions with potentially lifelong impacts on health and disease. Because MPB and TCS are commonly used as bactericides or fungicides, these chemicals have the potential to modify the microbiota, which, in turn, may influence human health. However, little direct evidence has been reported to suggest any interplay between environmental chemical exposure and the microbiome in human health, especially in adolescence in humans or in animal models exposed to chemical doses relevant to humans.

Herein, we employed a rodent model to determine whether exposure to chemicals found frequently in personal care products, i.e., DEP, MPB, TCS, and their mixture, administered at doses that result in urinary biomarker levels comparable to humans [20], affect the diversity of the gut microbiome at two developmental stages. This study stems from a parent study on windows of susceptibility to environmental chemical exposure on mammary gland development; therefore, only female rats were included in this investigation. We utilized the resources and animals of the parent study and collected fecal samples from adolescent (postnatal day (PND) 62) and adult (PND 181) female rats. This proof-of-principle study was designed to examine the potential impact of low-dose environmental toxicants on the composition of the gut microbiome.

## Results

### The gut microbiota in SD rats

16S ribosomal RNA (rRNA) gene PCRs were performed on 150 Sprague-Dawley (SD) rat fecal samples, 100 PND, 62 samples and 50 PND, 181 samples. In total, 17 samples (3 in OIL, 6 in DEP, 4 in MPB, 1 in TCS, and 3 in mixture (MIX)) were excluded because they showed no or poor DNA amplification. The rats for each treatment groups were housed in multiple cages to minimize the cage effect. We used a dual-barcoding sequencing approach to obtain high-quality sequencing data while minimizing the sequencing cost (illustrated in Additional file 1: Figure S1A). From a single Illumina MiSeq 250 × 2 pair-end sequencing run, and we generated ~3.7 million merged high-quality reads (quality score >30 at any position of the single read). After splitting by the barcodes, we obtained ~30 k high-quality reads per sample on average. The technical repeats were used to validate the reproducibility of the 16S rRNA gene sequencing. The taxonomy assignment showed that the mean correlation among the quadruplicates was within the range of 0.991 to 0.994 from phylum to genus level (Additional file 1: Figure S1B), while the mean correlation across all samples was 0.79 at the phylum level and 0.63 at the genus level.

**Fig. 1:**
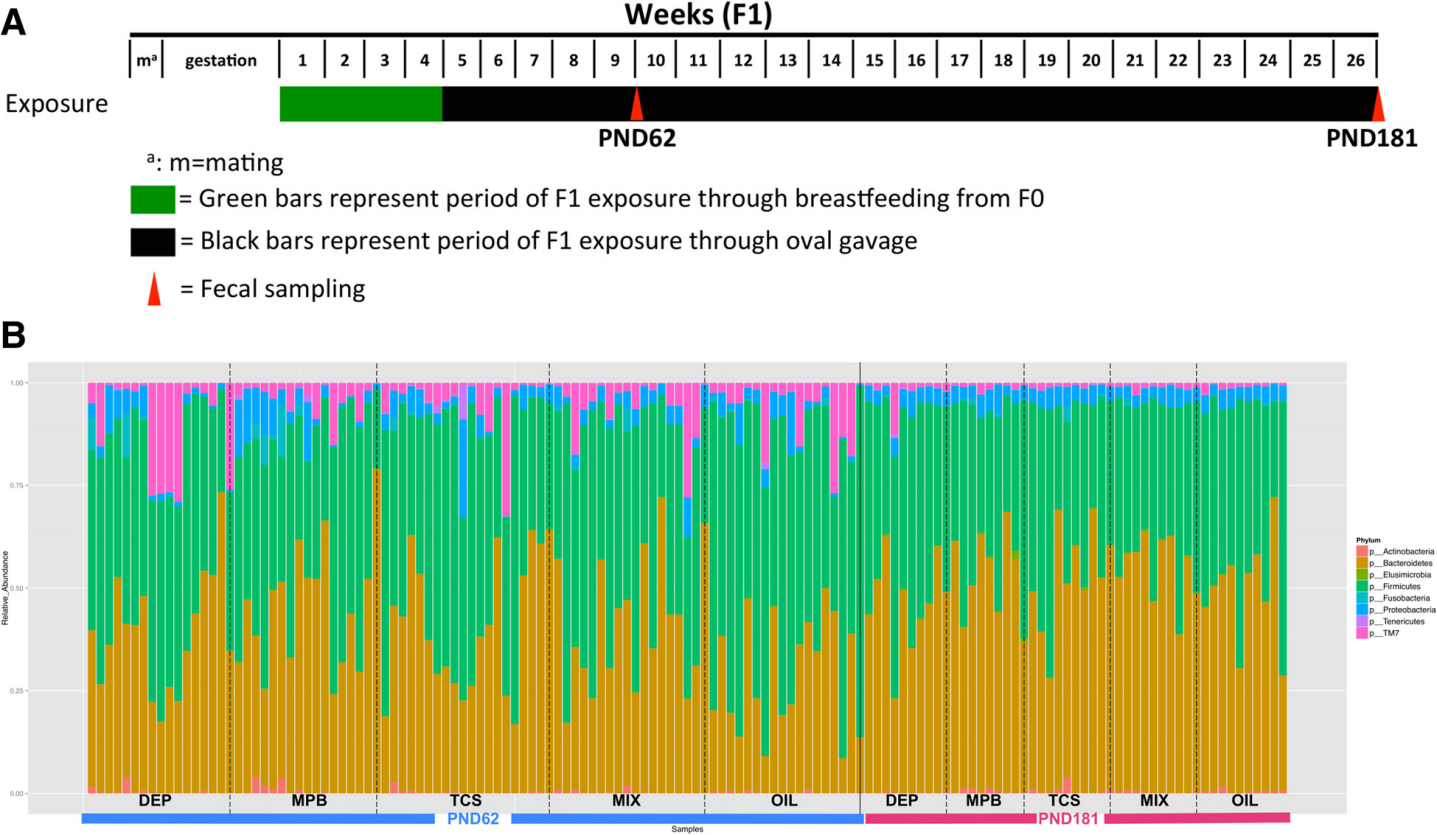
Composition of the rat microbiota at the phylum level. Samples were divided by ages (PND 62 or PND 181) and treatments. *OIL* oil control, *DEP* diethyl phthalate, *MPB* methylparaben, *TCS* triclosan, *MIX* mixture of equal quantities of the three environmental chemicals. **a** Illustration of the timeline of the environmental chemical treatments and fecal sample collection. **b** The bar plots present the relative abundance of phylum of each individual sample within each treatment group

Female SD rats were exposed to three chemicals (DEP, MPB, and TCS) and their mixtures as well as the vehicle alone (olive oil) from birth to adulthood (PND 1–181). The fecal droppings were collected from individual rat at two time points, PND 62 and PND 181. The experimental scheme is illustrated in Fig. 1a. Oral doses were selected to recapitulate human exposure levels; the urinary biomarker levels in our experiments were comparable to those observed in the US population [20, 21]. The unique feature of our study is the extreme low dose of exposure; these doses were between 1000 and 10,000-fold less than the reported NOAEL (no observed adverse effect level). Figure 1b shows the bacterial composition and the relative abundance at the phylum level of each individual sample grouped by treatment at these two time points. The difference with respect to chemical and time was apparent. Additional file 1: Figure S2 presents the bacterial composition and the relative abundance of individual samples at family levels. Consistent with previous studies [22], the dominant phyla in the rat gut microbiota were *Firmicutes*, *Bacteroidetes*, *Proteobacteria*, and *TM7.*

**Fig. 2:**
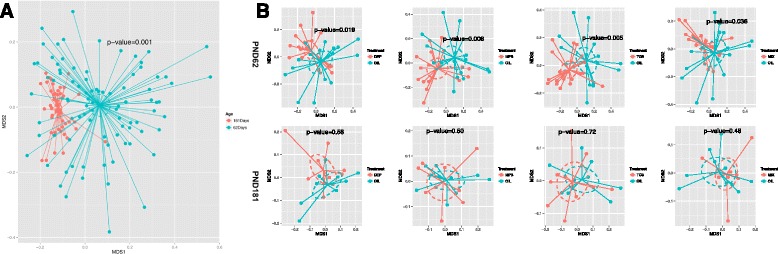
Comparison of overall microbiota from rats exposed to varied environmental chemical using nMDS ordination. The Bray-Curtis distance matrices generated from taxa composition and relative abundance at genus level were visualized in nMDS plot. The ellipses were drawn to represent the standard error. The texts of the group names were positioned at the center of each group. The significance of the dissimilarity of overall microbiota between two groups was tested using PerMANOVA. **a** Comparison of overall microbiota between PND 62 and PND 181. **b** Comparison of overall microbiota between each treatment and oil control at PND 62 (*upper panel*) or at PND 181 days (*lower panel*)

Comparing PND 62 to PND 181, we observed a significant reduction in relative abundance of *Firmicutes* (41 vs. 47 %; *p* = 0.005) and *TM7* (1 vs. 6 %, *p* < 0.001) and increase in *Bacteroidetes* (48 vs. 37 %, *p* < 0.001), regardless of the exposure. There was considerable separation of the overall microbiota diversity between the fecal samples collected at these two time points (*p* = 0.001, by permutational multivariate analysis of variance (PerMANOVA) test) (Fig. 2a); the mean sample-to-sample dissimilarity of gut microbiota was much higher at PND 62 than at PND 181 (Fig. 2a and Additional file 1: Figure S3A). Compared to PND 62, the samples collected at PND 181 showed higher bacterial community richness and lower variability within each treatment group (Additional file 1: Figure S3B).

### Chemical-induced changes of gut microbiota in SD rats

When comparing the chemical-treated to control rats, at phylum level, a significant reduction in abundance of *Firmicutes* (47 vs. 60 %, *p* = 0.004) and an increase of *Bacteroidetes* (41 % vs. 29 %, *p* = 0.003) were observed at PND 62 but not at PND 181 (Fig. 1b, Additional file 1: Figure S2). The *Firmicutes/Bacteroidetes* ratio, a metrics that is positively associated with obesity in mammals [23], was significantly lower in every chemical-treated group than the control at PND 62. Moreover, the exposed rats exhibited distinct microbiota from the control rats at PND 62 (Fig. 2b); the PerMANOVA test using the taxonomic composition at the genus level revealed significant separation of microbiota diversity in rats treated with DEP, MPB, TCS, and MIX from that of the controls (*p* = 0.02, 0.006,0.005, and 0.04, respectively). None of these treatments resulted in significant changes in the microbiota diversity at PND 181. No changes of average community richness were observed in any treatment group at either time point (Additional file 1: Figure S3B). We further compared the overall microbiota dissimilarity between the treatments (Additional file 1: Figure S4), and the PerMANOVA tests (Additional file 1: Table S1) suggested there were no significant differences.

**Fig. 3:**
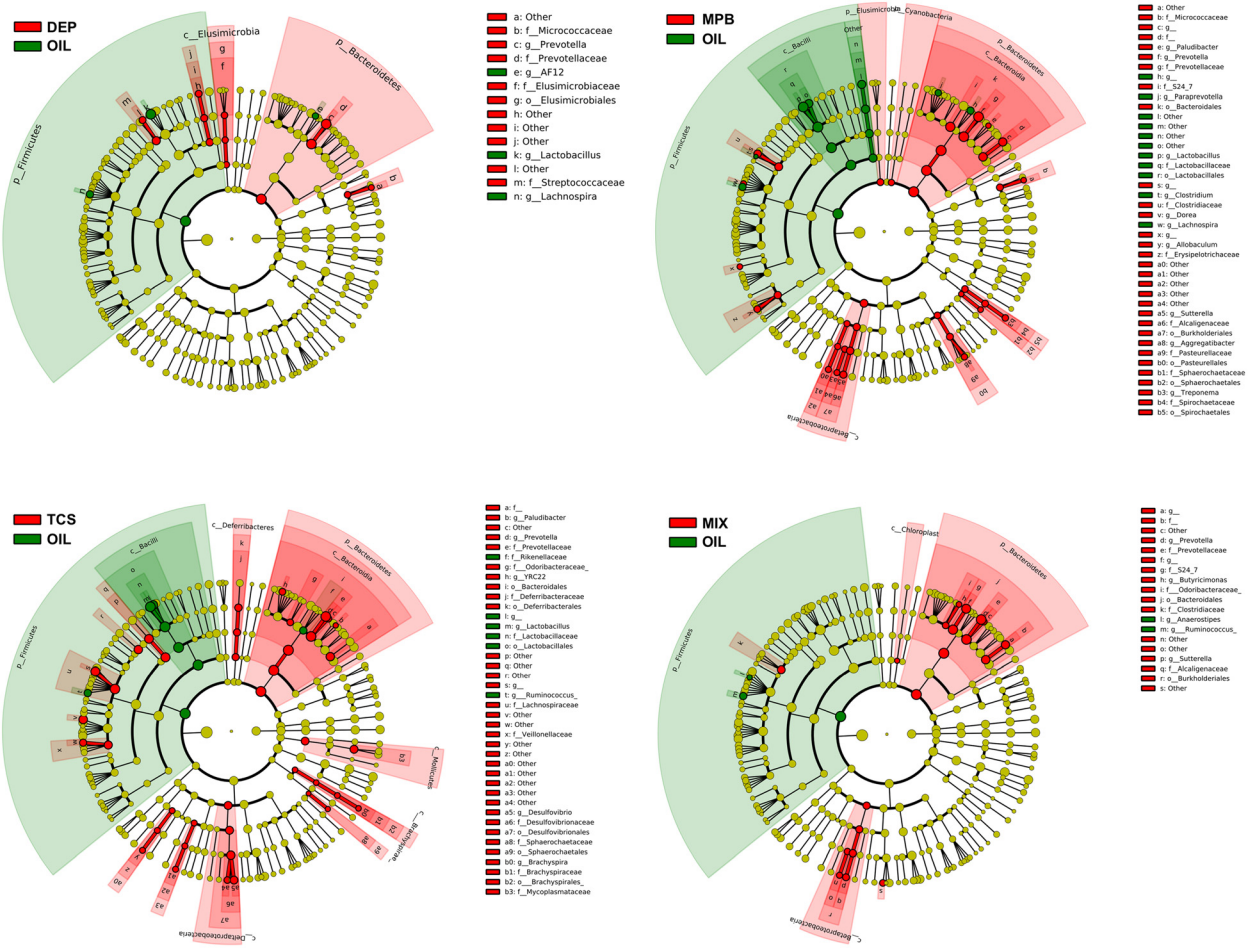
Taxonomic representation of statistically and biologically consistent differences between each environmental chemical treatment and controls at PND 62 rats. Cladogram plots present the LEfSe results on gut microbiome of environmental chemical-treated rats and controls at PND 62. Differences are represented in the color for the most abundant class (*red* indicating increase, *green* indicating reduction). Each circle’s diameter is proportional to the taxon’s relative abundance

We performed the linear discriminant analysis (LDA) effect size (LEfSe) analysis to compare the bacterial composition from phylum to genus levels between the chemical-treated and control groups. Results from PND 62 rats are shown in Fig. 3. At the phylum level, *Bacteroidetes* were increased and *Firmicutes* were reduced in all treated rats. Within the *Bacteroidetes* phylum, all treatments resulted in an increase in abundance of *Prevotella* species at the genus level (Fig. 3). Interestingly, in comparing the taxa composition associated with the three individual chemicals and their mixture, MPB and TCS, the two chemicals most commonly used as antimicrobial agent, showed similar microbiome shifts. TCS resulted in increase of *Lachnospiraceae,* which was also the major taxon increased in newborn mice exposed to low-dosage antibiotics in previous studies [9]. Compared to both MPB and TCS, DEP treatment showed more modest microbiome shifts. However, at the family level, the increases of *Streptococcaceae* and *Elusimicrobiaceae* were only observed in the DEP-treated animals, not in the MPB, TCS, or MIX groups. In addition, all treatments showed a reduction of *Lactobacillus*. The *Betaproteobacteria* at the class level was increased in the MPB or mixture groups, but not in the DEP- or TCS-treated rats. Importantly, no taxon was found to significantly differ by chemical exposure in the fecal samples collected at PND 181.

Using the conventional criterion of 97 % sequence similarity (equal to the species level), we clustered the sequencing reads and identified a total of 2980 operational taxonomic units (OTUs). Compared to their corresponding controls, the treatment groups at PND 62 resulted in more differential OTUs (*p* < 0.05, false discovery rate (FDR) adjusted) than at PND 181 (Additional file 1: Table S2). At PND 62 (Additional file 1: Figure S5), samples from the DEP and MPB treatment groups showed an increase in OTUs from *Fusobacteria* (*Fusobacterium* and *Leptotrichia* genera); this was not apparent in samples from the TCS- or MIX-treated groups. We also observed an increase in OTUs from *Bacteroidetes/Prevotella* genus with DEP, MPB, or TCS treatment along but not with MIX. In comparison to the controls, we identified varying OTUs with DEP, MPB, TCS, MIX treatments (9, 11, 4, and 5, respectively, *p* < 0.05, FDR adjusted; Additional file 1: Table S2); these OTUs correspond to coverages of ~25, 28, 11, and 6 % of the total bacterial population in the controls. In agreement with our observation at the genus level, environmental chemical treatment increased the relative abundance of OTUs from the *Prevotella* genus and reduced the relative abundance of OTUs from the *Lactobacillus* genus. However, at PND 181, only three OTUs were statistically significant (*p* value <0.05), covering 1 % of the population in the MIX treatment group, while none were found in the other treatment groups.

The mean bodyweight of the experimental rats was calculated at PND 62 and PND 181; the results are presented in Table 1. At PND 62, the mean bodyweight of the exposed rats was consistently lower than that of the controls; however, the reduction was small and not all *groups* reached statistical significance. For example, the MPB-treated rats had a mean bodyweight of 199.4 g compared to 209.4 g for the controls; the 10-g difference (*p* = 0.0083) represents a bodyweight reduction of <5 %, with unknown health consequences. At PND 181, the reduction in the mean bodyweight was much less prominent; although the mean bodyweight in all treated groups was lower than the controls, the reduction was 1–3 %.

**Table 1:** Mean bodyweights of environmental chemicals treated SD rats at PND 62 and PND 181

Treatment	PND 62			PND 181		
	Mean bodyweights (g)	Standard errors	*p* value*	Mean bodyweights (g)	Standard errors	*p* value
DEP	202.9	1.9	0.046	322.0	4.5	0.13
MPB	199.4	2.5	0.0083	327.2	5.3	0.39
TCS	206.5	2.7	0.43	328.5	4.8	0.46
MIX	207.2	2.7	0.56	331.0	3.1	0.63
OIL	209.4	2.5	Reference	334.5	6.3	Reference

*p value from Student’s *t* test

## Discussion

Humans are commonly exposed to a broad spectrum of environmental chemicals at a wide range of doses; concern over such exposure continues to rise because of suspected or potential adverse health effects. A recent Centers for Disease Control and Prevention report indicates that all Americans harbor significant levels of many chemicals in their bodies, many are universally detected and many have concentration levels above the part-per-billion level [11]. There is a small but growing body of evidence supporting a role for such exposure in metabolic dysregulation, exhibited as body size changes [13, 24–26], insulin resistance, or altered thyroid hormone levels [15, 27].

In the meantime, the gut microbiome has emerged as a key player in human metabolism [17, 28, 29]. It is thus reasonable to hypothesize that the exposure to environmental chemicals may modify the gut microbiome and ultimately influence human health. A recent study by Narrowe et al. showed the low-level triclosan exposure altered the gut microbiome of the fathead minnow [30]. For this study, we selected phthalates, parabens, and triclosan for investigation because they are ubiquitous in the human environment as well as antimicrobial activities. Cho et al. demonstrated that exposure of low-dose antibiotics in mice resulted in substantial taxonomic changes in the microbiome and associated changes in the metabolism of carbohydrates to short-chain fatty acids, as well as the regulation of hepatic metabolism of lipids and cholesterol [9]. Schubert et al. also reported that the different structural shifts in the mouse microbiome resulting from various antibiotic exposures altered susceptibility to *Clostridium difficile* colonization [31]. We therefore postulate that low-dose exposure to these prevalent environmental chemicals may also modulate the composition of the gut microbiome. Results from this study demonstrate such capacity when exposed early in life, even at very low doses that are comparable to the human exposure.

Several studies supported by the Human Microbiome Project report differences in microbiota between children and adults [32–36]. Studies also suggest that variation of microbiota is highest during childhood and gradually decreases with age [33, 34, 37]. Similar to the findings in human studies, samples from the adolescent rats (PND 62) in our experiment also showed distinctive overall microbiota compared to the adult rats (PND 181), with higher relative abundance of *Firmicutes* and lower relative abundance of *Bacteroidetes*. However, how these two time points translate into human developmental stages is not totally clear; thus, caution is warranted in interpreting our findings. Compared to humans, rats have an abbreviated and accelerated childhood; they develop rapidly during infancy and become sexually mature at about 6 weeks of age (PND 42) or at 40 to 60 days [38]. Humans, on the other hand, develop slowly and do not reach puberty until the age of 11–12 years [39]. In rats, the period of PND 55–66 often represents the adolescent period and the transition to adulthood begins after the eighth week of postnatal life [40].

Compared to the adult rats, the gut microbiome of adolescent rats in our experiment showed lower taxon richness but higher variance within sample and higher sample-to-sample dissimilarity. Furthermore, our results revealed that all three chemicals, at levels comparable to those humans would likely encounter, significantly altered the overall microbiota diversity and resulted in more prominent changes at multiple taxon levels in the adolescent rats. However, the effects were diminished in the adult rats. We also observed a subtle yet consistent reduction in the bodyweight of the young rats, which corresponded with the observed shift in gut microbiota, in particular the reduced *Firmicutes/Bacteroidetes* ratio. It is known that the gut microbiota is continually subject to a wide variety of perturbations, including various environmental factors [41, 42]. However, even with these insults, the gut microbiota is generally stable over time due to the resilience of commensal microbes to survive under continuous challenge. In our experiment, the rats underwent treatment continuously from birth. Although the gut microbiome showed significant changes by treatment at the earlier age (adolescent), the gut microbiota appeared to be able to converge or recover at a later (adult) stage when the environmental-induced changes were minimized. These data suggest that the commensal gut microbiota can develop resistance to the low-dose environmental chemical exposure during development.

Low-dose antibiotic exposure, likely from environmental sources, showed no significant effect on the bacterial diversity within the samples [9]. Similar to antibiotics, in our study, exposure to low-dose environmental chemicals did not reduce the biodiversity of the gut microbiota. Nevertheless, environmental chemical exposures at such low doses were still capable of altering the overall composition of the gut microbiota. We observed an increase in abundance of *Lachnospiraceae* in TCS-treated rats, similar to that previously described in low-dose antibiotic-treated rodents [9]. However, in contrast to the increased *Firmicutes/Bacteroidetes* ratio in antibiotic-treated mice, we noted an increase in the relative abundance of *Bacteroidetes* compared to *Firmicutes* in the exposed adolescent rats, correlated with an observed reduction in bodyweight. These results may suggest different functional mechanisms between the low-dose antibiotics and the environmental chemicals used in this study; it may also reflect the inherent differences between rodents and humans. Such complexity of the association between environmental exposure and obesity-related outcomes has been recognized [43]. However, results on *Firmicutes/Bacteroidetes* ratio and obesity from other studies are inconsistent and warrant further validations [44, 45].

The combined effect of multiple exposures is rarely considered in environmental investigations. Considering that many chemicals, like the ones investigated in this study, are commonly used together in personal care products, it is unlikely that we encounter environmental chemicals one at a time. Recent investigations into the low-dose mixture effects of endocrine disruptors [46] demonstrate not only additive but also synergistic or antagonistic effects [47]. In the current study, treatment with mixture resulted in a distinct microbiome shift that differed from that of individual chemical or a simple additive effect, suggesting possible biological interactions of these chemicals.

The major limitation of the investigation is the lack of health-related outcomes in the animal model. This study stemmed from a parent study on the effects of environmental chemicals on normal mammary gland development, in which established microbiome-related outcomes, such as obesity or colitis, were not induced. Nevertheless, by utilizing the resources of the parent study, we were able to demonstrate that commonly used environmental chemicals have the capacity to change gut microbiome composition at a dose that is comparable to human exposure in the US population. More importantly, our results suggested that the early-life exposure resulted in more observable changes in the microbiome composition. A recent study using a rodent model demonstrated that altering the gut microbiota during a critical developmental window might have lasting metabolic consequences [48]. We are in the process of conducting the follow-up studies on the potential health outcomes. Another limitation is the use oral gavage as the route of exposure. Given the ubiquitous nature of the study compounds, human exposure is likely to involve multiple routes, via skin absorption, ingestion, and inhalation [49]. Several studies showed that breast milk during lactation also contains these three chemicals or their metabolites [50, 51]. While is it almost impossible to recreate exact human exposure scenario, we tried to resolve this issue by calibrating the exposure dose to achieve similar urinary biomarker concentrations between rats and humans. Gavage is preferred over other routes of exposure for environmental chemicals when very low doses are used [52]. It is difficult to ascertain the true intake when chemicals are mixed into food or drinking water ad libitum. Although gavage does not perfectly represent a model of human dietary exposure, this route has been employed for numerous studies assessing potential carcinogenic hazards [53]. Lastly, because the parent study was designed to investigate the underlying mechanisms of environmental exposures and breast cancer, only data on female rats were available so that any gender-specific effects on microbiome cannot be addressed in this investigation.

## Conclusions

In summary, our study provides the first evidence that postnatal exposure to commonly used environmental chemicals at levels comparable to human exposure is able to alter the gut microbiota in a rodent model. These findings enhance our understanding on the impact of environmental toxicants on the composition of gut microbiome and, potentially, on the human health.

## Methods

### Animal study

All animal study procedures were performed at the Cesare Maltoni Cancer Research Centre/Ramazzini Institute (CMCRC/RI) (Bentivoglio, Italy). The experiment was conducted following the rules established by the Italian law regulating the use and human treatment of animals for scientific purposes (Decreto legislativo N. 26, 2014. Attuazione della direttiva n. 2010/63/UE in materia di protezione degli animali utilizzati a fini scientifici. - G.U. Serie Generale, n. 61 del 14 Marzo 2014). Before starting the experiment, the protocol was examined by the Ethical Committee of Ramazzini Institute for approval. The protocol of the experiment was also approved and authorized by the ad hoc commission of the Italian Ministry of Health and the Mount Sinai IACUC. Because the original study focuses on windows of susceptibility in breast cancer, only female SD rats, belonging to the colony used in the laboratory of the CMCRC for over 40 years, were used. The experimental animals (F1) received the treatment from birth (PND 1) through milk of dams (F0) exposed to environmental chemicals from parturition. After weaning, the female offspring (F1) were exposed through gavage three times a week until euthanization at PND 181.

The timeline of the experimental animal treatment and fecal sample collection is shown in Fig. 1a. The breeder animals (F0) were weighed weekly, starting after parturition, and the dose to be administered during lactation was calculated on the basis of the weekly weight. All the pups (F1) were housed with their dam (F0) until weaning; then, they were separated and identified by ear punch; each litter contributed to the study with one female pup. The animals were randomized in the different groups of treatment in order to have minimal differences in bodyweight among them, with a standard deviation of no more than 10 % from the average. They were housed in Makrolon cages (cm 41 × 25 × 15) at two or three per cage, with a stainless steel wire top and a shallow layer of white firewood shavings as bedding. All animals were kept in a single room at 23 ± 3 °C and at 40–60 % relative humidity. Lighting was artificial and the light/dark cycles were tended to be 12 h each. The F1 generation received the treatment through breast milk from PND 1 to PND 28 (weaning). Young females were then separated from their mothers and weighed individually every week; they were dosed by gavage following the protocol, based on the weekly mean bodyweight of each group. The animals were given the same standard “Corticella” pellet diet (Piccioni Laboratory, Milan, Italy) for both breeders and offspring; both feed and tap water were available ad libitum. Feed and tap water were periodically analyzed to exclude biological and chemical contamination (mycotoxins, pesticides, arsenic, lead, mercury, selenium). During the experiment, the mean daily water and feed consumption were measured per cage; bodyweights were individually measured once a week for the first 13 weeks and every two weeks until the end of the experiment. The experimental protocol is outlined in Table 2.

**Table 2:** Experimental plan of environmental chemicals treatment and stool sampling

Group	Compound (dose in mg/kg bw)^a^	Animals at start	Monitored animals	
		No.	PND 62	PND 181
I	DEP (0.1735)	20	20	10
II	MPB (0.1050)	20	20	10
III	TCS (0.050)	20	20	10
IV	MIX (mixture of DEP + MPB + TCS)	20	20	10
V	OIL	20	20	10
Total		100	100	50

*DEP* diethyl phthalate, CAS 84-66-2, *MPB* methylparaben, CAS 99-76-3, *TCS* triclosan, CAS 3380-34-5), *MIX* mixture of DEP + MPB + TCS in equal quantities, *OIL* olive oil control, vehicle alone

^a^Animals were treated from PND 1 to PND 181. The newborns (F1) were first exposed to chemicals postnatally through milk from the exposed dams, from birth to weaning (PND 28). After weaning, these pups were exposed through oral gavage three times a week, from PND 28 to PND 181. Each compound was administered in olive oil as vehicle by gastric intubation (gavage), starting with 0.5 ml of olive oil from 4 to 9 weeks of age, and then with 1 ml once adult

### Tested compounds

Three chemicals and their mixture were tested along with a control group exposed to vehicle only. These chemicals, i.e., diethyl phthalate (CAS # 84-66-2, lot # STBB0862V, 99 % purity), methylparaben (CAS # 99-76-3, lot # BCBG0852V, 99 % purity), and triclosan (CAS # 3380-34-5, lot # 1412854 V, 97 % purity) were supplied in plastic containers by Sigma-Aldrich (Milan, Italy). Olive oil, supplied in glass bottles (Montalbano Agricola Alimentare Toscana, Florence, lot # 111275, Italy), was used as the vehicle to prepare all dosing solutions. Olive oil was tested to be free of the tested chemicals. The solutions were prepared every week using glass pipettes, stored in glass bottles, continuously stirred throughout the study, and kept at room temperature and in the dark; the stability of the solutions was confirmed by gas chromatography-mass spectrometry (GC-MS) (Neotron Laboratory, Modena, Italy). Precautions were taken to minimize any plastic contamination: the compounds were administered using a 5-ml glass syringe, and the biological samples were collected in polypropylene vials.

### Dosage of chemical exposure

With knowledge of the toxicokinetic properties of the tested chemicals, we determined oral doses of DEP, MPB, and TCS that resulted from urinary biomarker concentrations comparable to those reported for the US population [20, 21]. Specifically, the oral doses were: NOAEL/10,000 for DEP and MPB; NOAEL/1000 for TCS, resulting in 0.1735, 0.105, and 0.05 mg/kg/day final doses, respectively. The mixture solutions were prepared by mixing the three chemicals at the selected dose. The control group received gavage with olive oil alone, which was the vehicle selected.

The experiment started with 100 dams (F0), 20 in each experimental group (three chemicals, one mixture and control). Their newborns (F1) were first exposed to chemicals postnatally through milk from the exposed dams, from birth to weaning (PND 28). After weaning, these pups were exposed through oral gavage three times a week (on Monday, Wednesday, and Friday), at similar times of the morning, and adjusted weekly to maintain a constant dose level in terms of bodyweight, from PND 28 to PND 181.

### Fecal collection

Fecal samples were collected from all animals of the F1 generation (20 from each test compound) at PND 62. Afterwards, due to budgetary constraints, 50 rats, 10 from each test compound, were randomly selected and carried on with the chemical treatment until PND 181 when the fecal samples were collected. After oral gavage with the test chemicals, each animal was single-caged for at least 4 h in order to avoid contamination of fecal droppings from other animals. About 2–3 droppings from each animal were collected and preserved in cryovials on an ice bed. Forceps were used for collecting droppings, which were washed and cleaned using sterile water and 1 % sodium bicarbonate between each sampling to avoid cross contamination. The cryovials were then stored at −20 °C until shipment to the Icahn School of Medicine at Mount Sinai.

### Fecal DNA extraction and processing

Rat fecal DNA was extracted using the Qiagen DNA Stool Mini Kit following the protocol from the manufacturer (Qiagen, Valencia, CA). Total DNA concentration was determined by Qubit 2.0 Fluorometer (Life technologies, Norwalk, CT). The phylogenetically informative V3–V4 region of 16S rRNA gene was amplified using universal primer 347F/803R [54, 55]. We designed a dual-barcoding approach to label the 16S rRNA gene amplicons from each sample (illustrated in Additional file 1: Figure S1A). Briefly, the 6-mer barcodes were attached on the 5′ends of both forward and reverse PCR primers so that 16S rRNA gene PCR amplicons from each sample contained a unique dual barcode combination. PCR primers were designed against conserved sequences to amplify the flanking variable 16S rRNA gene regions. The primers were synthesized by IDT (Integrated DNA technology, Coralville, IA), and the sequences are shown in Additional file 1: Table S3.

### 16S rRNA gene sequencing and data analysis

The 16S rRNA gene was then amplified by PCR with double-barcoded primer pairs. The integrity of the amplicons was verified by agarose gel electrophoresis. The resulting ~460-bp sized amplicons were pooled and then sequenced with the Illumina MiSeq paired-end sequencing platform. The 2 × 250 pair-end sequence fastq data were merged. After removal of the merged sequencing reads with a length of <400 or the quality score of < Q30 at more than 1 % of bases, all sequencing reads were split by barcode and trimmed of primer regions using CLC Genomic workbench 6. Quadruplicate measurements of one sample and duplicate measurements of three samples were processed and sequenced using different barcodes and batches to test the sequencing reproducibility. The filtered and trimmed high-quality reads were further processed by QIIME 1.7.0 [56]. We used the command *pick_de_novo_otus.py* with the defaulted cutoff = 97 % to cluster of nearly identical sequencing reads as an OTU using Uclust. Representative sequences for each OTU were aligned using PyNAST. The program further assigned taxonomy with the Uclust consensus taxonomy assigner and filtered the alignment to remove positions, which are all gaps, and specified as 0 in the lanemask. Finally, the program built a biom-formatted OTU table. Using Chimera Slayer [57], chimera sequences arising from the PCR amplification were detected and excluded from the aligned representative sequences and the OTU table. All non-singleton OTUs were retained for performing the log likelihood ratio test (QIIME command group_significance.py using g_test statistics) to further identify significant differential OTUs between each treatment and the normal controls. The resulted *p* values were adjusted by the FDR methods.

The overall microbiome dissimilarities among all samples were accessed using the Bray-Curtis distance matrices [58, 59]. Non-metric multiple dimensional scaling (nMDS) were used to visualize the dissimilarities. The PerMANOVA procedure [60, 61], with the maximum number of permutations = 999, was performed to test the significance of the overall microbiome differences between the gut microbiota grouped by PNDs and chemical treatments. The PerMANOVA procedure using the [Adonis] function of the *R* package *vegan* 2.0–5 [62] partitions the distance matrix among sources of variation, fits linear models to distance matrices and uses a permutation test with pseudo-*F* ratios to obtain the *p* values. The diversity within each microbial community, so-called alpha-diversity, was calculated using the Shannon Index as metric and represented the measure of the diversity at the genus level [63, 64]. Using the LEfSe method [65], we further selected the microbiome features significantly associated to PNDs and environmental chemical treatment at various taxonomic ranks.

## Abbreviations

DEP, diethyl phthalate; LEfSe, linear discriminant analysis (LDA) effect size; MIX, mixture; MPB, methylparaben; OTU, operational taxonomic units; PerMANOVA, permutational multivariate analysis of variance; PND, postnatal day; SD, Sprague-Dawley; TCS, triclosan

## Additional file

Additional file 1: Table S1. The significance of the overall microbiota differences between treatment groups by PerMANOVA (permutational multivariate analysis of variance) procedure. **Table S2.** List of differential OTUs by treatment at 62 days and 181 days. **Table S3.** The sequences of dual-barcoding 16S PCR primers. **Figure S1.** Multiplex double-barcoding 16S rRNA sequencing. 1A. Illustration of multiplex double-barcoding 16S rRNA sequencing. **Figure S2.** Profiles of the rat microbiota at the family level. **Figure S3.** Microbiota diversity in environmental chemical treatment and control groups. **Figure S4.** Comparison of overall microbiota from rats exposed to varied environmental chemical at PND 62 and PND 181 using nMDS ordination. **Figure S5.** The differential OTUs by treatment at PND 62. (PDF 4.93 mb)
