# Supplementary material for: Effect of postnatal low-dose exposure to environmental chemicals on the gut microbiome in a rodent model
Source: Microbiome. 2016 Jun 14;4:26. doi: 10.1186/s40168-016-0173-2 (PMC4906585; doi:10.1186/s40168-016-0173-2)
Supplement: Additional file 1: Table S1. — The significance of the overall microbiota differences between treatment groups by PerMANOVA (permutational multivariate analysis of variance) procedure. Table S2. List of differential OTUs by treatment at 62 days and 181 days. Table S3. The sequences of dual-barcoding 16S PCR primers. Figure S1. Multiplex double-barcoding 16S rRNA sequencing. 1A. Illustration of multiplex double-barcoding 16S rRNA sequencing. Figure S2. Profiles of the rat microbiota at the family level. Figure S3. Microbiota diversity in environmental chemical treatment and control groups. Figure S4. Comparison of overall microbiota from rats exposed to varied environmental chemical at PND 62 and PND 181 using nMDS ordination. Figure S5. The differential OTUs by treatment at PND 62. (PDF 4.93 mb) [file 40168_2016_173_MOESM1_ESM.pdf]

Supplementary Tables

Table S1. The significance of the overall microbiota differences between treatment groups by PerMANOVA (Permutational Multivariate Analysis of Variance) procedure. Test was performed with the maximum number of permutations = 999 and used the [Adonis] function of the *R* package *vegan* 2.0-5 partitions the Bray Curtis distance matrix among sources of variation, fits linear models to distance matrices and uses a permutation test with pseudo-F ratios to obtain the p-values.

PND62

Treatment	MPB	TCS	MIX	OIL
DEP	0.29	0.067	0.20	**0.019**
MPB		0.23	0.2	**0.006**
TCS			0.18	**0.005**
MIX				**0.039**

PND181

Treatment	MPB	TCS	MIX	OIL
DEP	0.17	0.39	0.064	0.57
MPB		0.41	0.23	0.5
TCS				0.71
MIX				0.5

Table S2. List of differential OTUs by treatment at 62 days and 181days. Fold changes were calculated using the mean taxa abundance of the treatment group divided by the control group. We performed the log likelihood ratio test (QIIME command group_significance.py using g_test statistics) to further identify significant differential OTUs between each treatment and the normal controls. The resulted *p*-values were adjusted by the FDR methods.

At 62 Days

Treatments	OTUs with FDR adjusted p-value <0.05				
	OTU ID	Taxonomy (family/genus)	Abundance%		Fold change
			Treated	Control	
DEP	171232	Lactobacillaceae/Lactobacillus	1.5	9.0	0.16
	1107027	Lactobacillaceae/Lactobacillus	6.9	1.3	5.5
	4397402	Lactobacillaceae/Lactobacillus	3.3	9.4	0.35
	137580	Lactobacillaceae/Lactobacillus	0.24	2.6	0.09
	47365	Lactobacillaceae/Lactobacillus	0.07	1.3	0.05
	2614328	Fusobacteriaceae/Fusobacterium	0.9	0.02	45.7
	291045	Ruminococcaceae/non-identified	0.26	1.3	0.2
	3878210	Peptostreptococcaceae/Peptostreptococcus	0.59	0.01	59.3
	4452538	Fusobacteriaceae/Fusobacterium	0.6	0.02	40
MPB	171232	Lactobacillaceae/Lactobacillus	1.1	9.0	0.12
	4397402	Lactobacillaceae/Lactobacillus	2.5	9.5	0.26
	579750	Non-identified	0.02	1.4	0.01
	137043	Lactobacillaceae/Lactobacillus	0.49	2.4	0.21
	47365	Lactobacillaceae/Lactobacillus	0.15	1.4	0.10
	4455767	Streptococcaceae/Streptococcus	0.89	0.03	29.5
	4297695	Campylobacteraceae/Campylobacter	0.82	0.03	32.6
	182016	Non-identified	0.31	1.4	0.22
	392918	Ruminococcaceae/non-identified	1.1	0.20	5.7
	524661	Bacteroidaceae/Bacteroides	1.0	0.17	6.2
	137580	Lactobacillaceae/Lactobacillus	0.98	2.5	0.4
TCS	579750	Non-identified	0.05	1.4	0.03
	176298	Non-identified	2.0	0.4	4.8
	291055	Prevotellaceae/Prevotella	1.4	0.2	7.1
	4397402	Lactobacillaceae/Lactobacillus	5.5	9.2	0.6
MIX	291045	Ruminococcaceae/non-identified	0.06	1.3	0.047
	579750	Non-identified	0.17	1.5	0.11
	29029	Ruminococcaceae/Ruminococcus	0.46	1.9	0.24
	279298	Non-identified	1.8	0.4	4.3
	326231	Ruminococcaceae/Ruminococcus	0.2	1.2	0.17

At 181 Days

MIX	316277	Non-identified	1.03	0.03	37
	176298	Non-identified	0.04	0.91	0.04
	261325	Ruminococcaceae/non-identified	0.98	0.08	12.6

Table S3. The sequences of dual-barcoding 16S PCR primers. The primer sequences include 2 bp linker (italicized) and 6 bp barcodes (bold) at the 5' end.

Primer ID	Sequence (5' to 3')
803R_b1	*AG***TCGACA**CTACCRGGGTATCTAATCC
803R_b2	*AG***GAACTC**CTACCRGGGTATCTAATCC
803R_b3	*AG***CTGTAG**CTACCRGGGTATCTAATCC
803R_b4	*AG***GACACG**CTACCRGGGTATCTAATCC
803R_b5	*AG***AGCGAG**CTACCRGGGTATCTAATCC
803R_b6	*AG***TCTCTA**CTACCRGGGTATCTAATCC
803R_b7	*AG***CGTGTC**CTACCRGGGTATCTAATCC
803R_b8	*AG***ATGCGT**CTACCRGGGTATCTAATCC
803R_b9	*AG***AACGCA**CTACCRGGGTATCTAATCC
803R_b10	*AG***ATTACC**CTACCRGGGTATCTAATCC
803R_b11	*AG***TGGTCA**CTACCRGGGTATCTAATCC
803R_b12	*AG***CCGTTT**CTACCRGGGTATCTAATCC
347F_b1	*AG***TCGACA**GGAGGCAGCAGTRRGGAAT
347F_b2	*AG***GAACTC**GGAGGCAGCAGTRRGGAAT
347F_b3	*AG***CTGTAG**GGAGGCAGCAGTRRGGAAT
347F_b4	*AG***GACACG**GGAGGCAGCAGTRRGGAAT
347F_b5	*AG***AGCGAG**GGAGGCAGCAGTRRGGAAT
347F_b6	*AG***TCTCTA**GGAGGCAGCAGTRRGGAAT
347F_b7	*AG***CGTGTC**GGAGGCAGCAGTRRGGAAT
347F_b8	*AG***ATGCGT**GGAGGCAGCAGTRRGGAAT
347F_b9	*AG***AACGCA**GGAGGCAGCAGTRRGGAAT
347F_b10	*AG***ATTACC**GGAGGCAGCAGTRRGGAAT
347F_b11	*AG***TGGTCA**GGAGGCAGCAGTRRGGAAT
347F_b12	*AG***CCGTTT**GGAGGCAGCAGTRRGGAAT

Supplementary Figures

S1. Multiplex double-barcoding 16S rRNA sequencing. 1A. Illustration of Multiplex double-barcoding 16S rRNA sequencing. Bacterial 16S DNAs were PCR amplified by universal primers. 8-bp barcodes were added at both forward and reverse primers. After PCR, 16S amplicons were pooled and sequenced. Based on the unique combination of the barcodes at both ends, the sequencing reads can be assigned to individual samples. **1B. Sequencing reproducibility.** The reproducibility of the 16S rRNA sequencing was validated using quadruplicate measurements. The bar plots present the taxa assignment and abundance of the quadruplicate measurements from phylum to genus level. At the same taxa level, different color stands for different taxa. The mean correlation among the quadruplicates was within the range of 0.991 to 0.994, while the mean correlation cross all samples is only 0.79 at phylum and 0.63 at genus.

S2. Profiles of the rat microbiota at family level. The bar plots present the taxa assignment and abundance of each individual samples grouped by age (PND 62 or PND 181) and treatments (OIL: oil control; DEP: Diethylphtalate; MPB: Methylparaben; TCS: Triclosan; and MIX: mixture of equal quantities of the three EDs).

S3. Microbiota diversity in environmental chemical treatment and control groups. S3A. The box plots present the mean and variance of the overall dissimilarity within groups by age or treatments. **S3B.** The box plots present the mean and variance of the alpha-diversity within groups by age or treatment.

S4. Comparison of overall microbiota from rats exposed to varied environmental chemical at PND62 and PND181 using nMDS ordination. The Bray-Curtis distance matrices generated from taxa composition and relative abundance at genus level were visualized in nMDS plot. The ellipses were drawn to represent the standard error. The texts of the group names were positioned at the center of each group.

S5. The differential OTUs by treatment at PND 62. Those OTUs were assigned to each genus and the logarithmized fold changes (treated/control) were shown in y-axis. The IDs of those OTUs Colors indicate the type of phylum. The size of the circle indicates the abundance of the OTU in control samples.

Figure S1

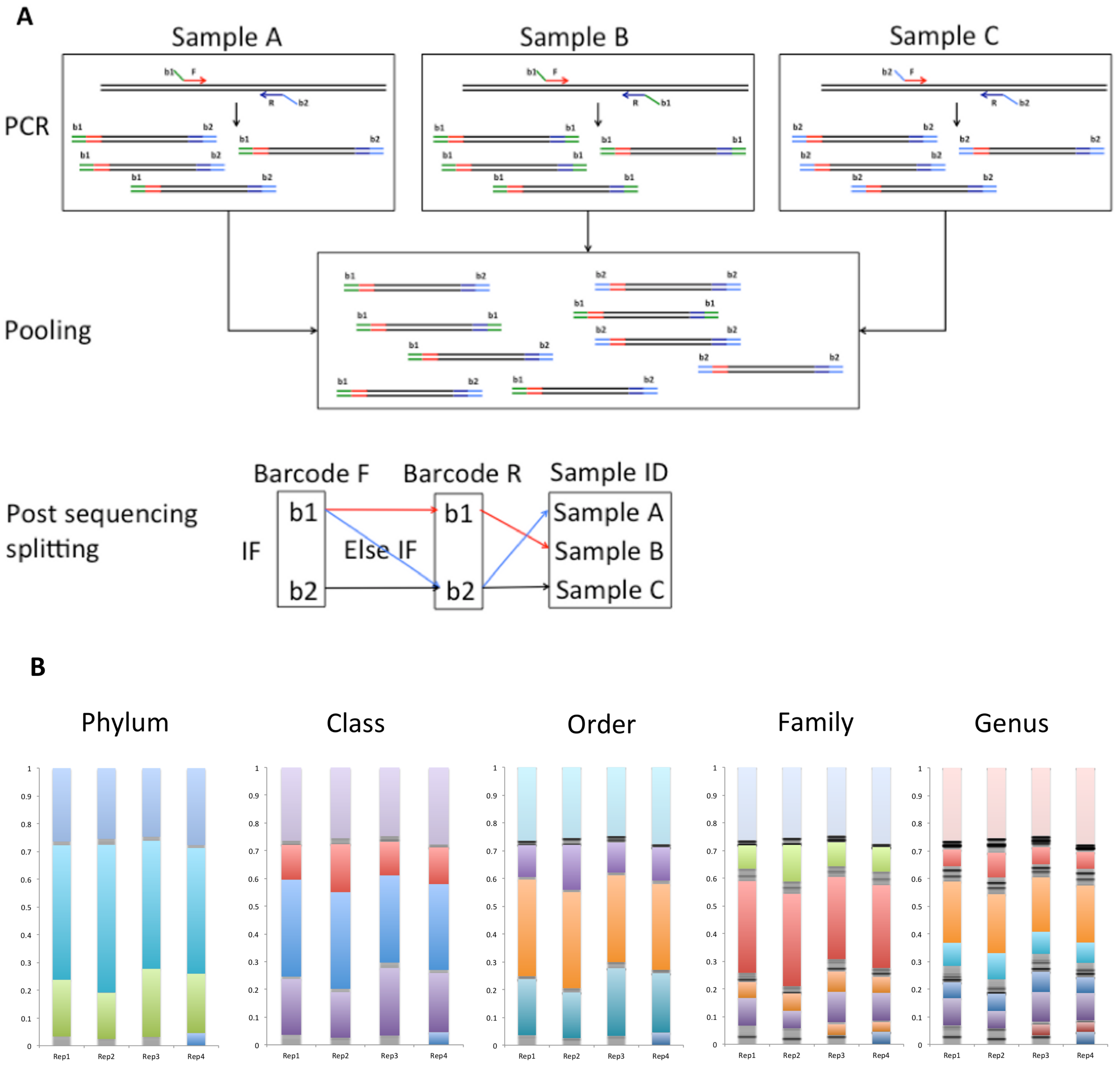

A
Sample A
Sample B
Sample C
PCR
Pooling
Post sequencing splitting
Barcode F
Barcode R
Sample ID
IF
Else IF
b1
b2
Sample A
Sample B
Sample C
B
Phylum
Class
Order
Family
Genus
Rep1
Rep2
Rep3
Rep4

Figure S2

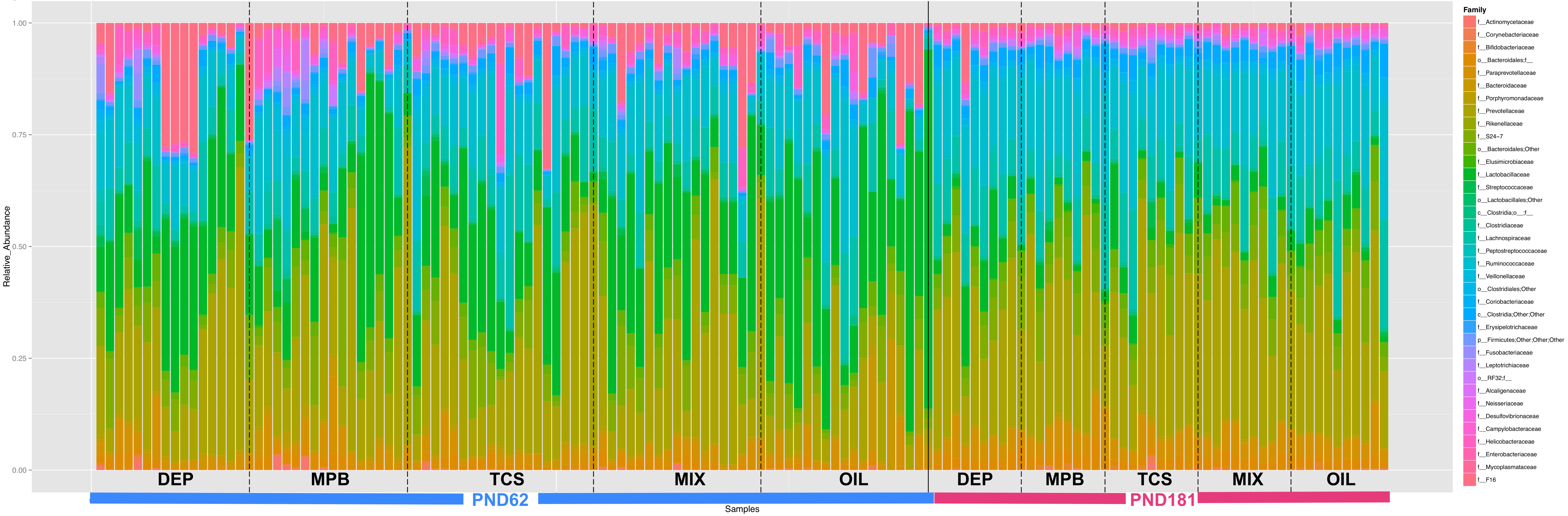

1.00
0.75
0.50
0.25
0.00
Relative_Abundance
DEP
MPB
TCS
MIX
OIL
DEP
MPB
TCS
MIX
OIL
PND62
PND181
Samples
Family
f__Actinomycetaceae
f__Corynebacteriaceae
f__Bifidobacteriaceae
o__Bacteroidales;f__
f__Paraprevotellaceae
f__Bacteroidaceae
f__Porphyromonadaceae
f__Prevotellaceae
f__Rikenellaceae
f__S24−7
o__Bacteroidales;Other
f__Elusimicrobiaceae
f__Lactobacillaceae
f__Streptococcaceae
o__Lactobacillales;Other
c__Clostridia;o__;f__
f__Clostridiaceae
f__Lachnospiraceae
f__Peptostreptococcaceae
f__Ruminococcaceae
f__Veillonellaceae
o__Clostridiales;Other
f__Coriobacteriaceae
c__Clostridia;Other;Other
f__Erysipelotrichaceae
p__Firmicutes;Other;Other;Other
f__Fusobacteriaceae
f__Leptotrichiaceae
o__RF32;f__
f__Alcaligenaceae
f__Neisseriaceae
f__Desulfovibrionaceae
f__Campylobacteraceae
f__Helicobacteraceae
f__Enterobacteriaceae
f__Mycoplasmataceae
f__F16

Figure S3

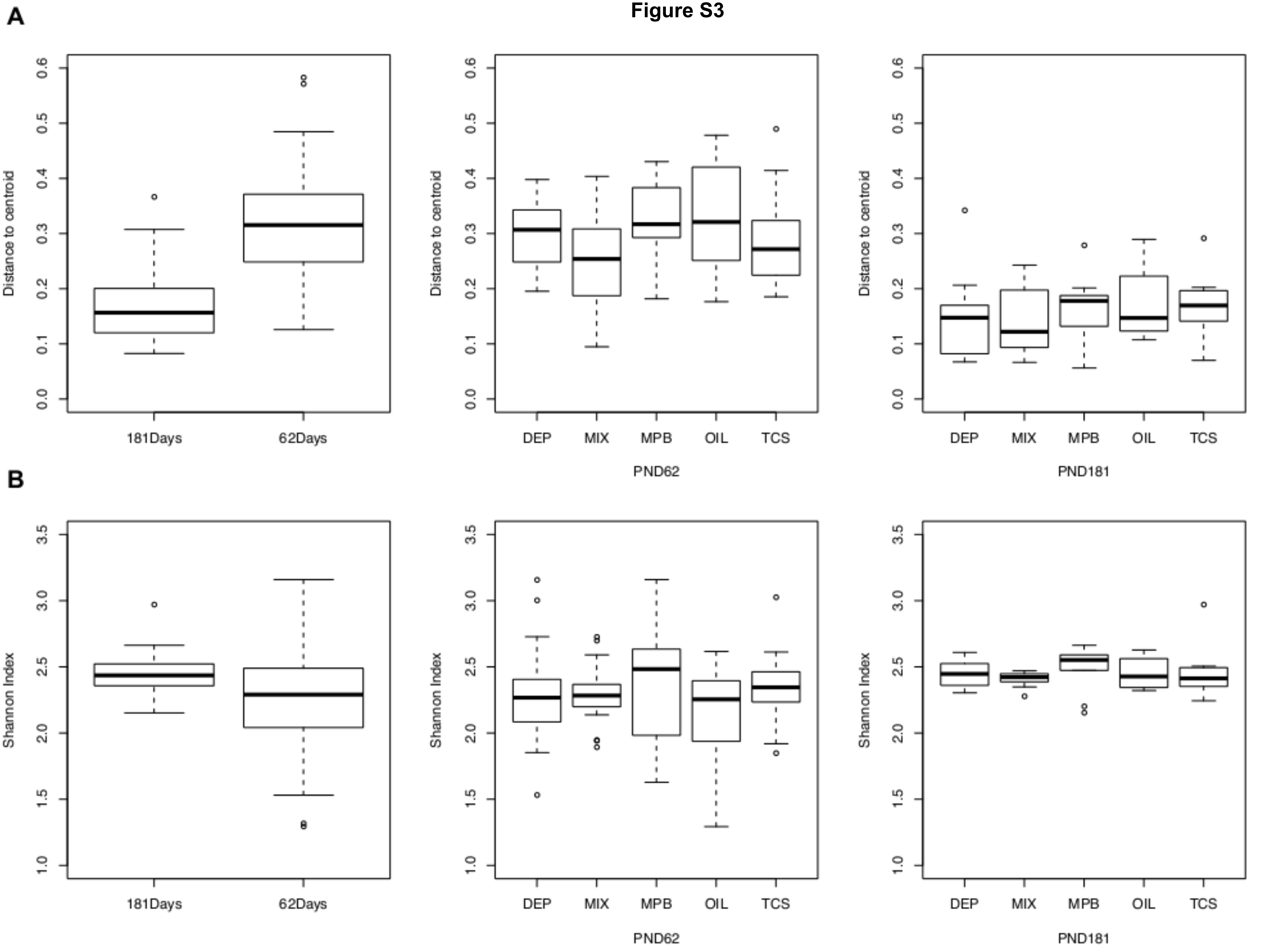
A
Distance to centroid
0.0
0.1
0.2
0.3
0.4
0.5
0.6
181Days
62Days
DEP
MIX
MPB
OIL
TCS
PND62
PND181
B
Shannon Index
1.0
1.5
2.0
2.5
3.0
3.5

Figure S4

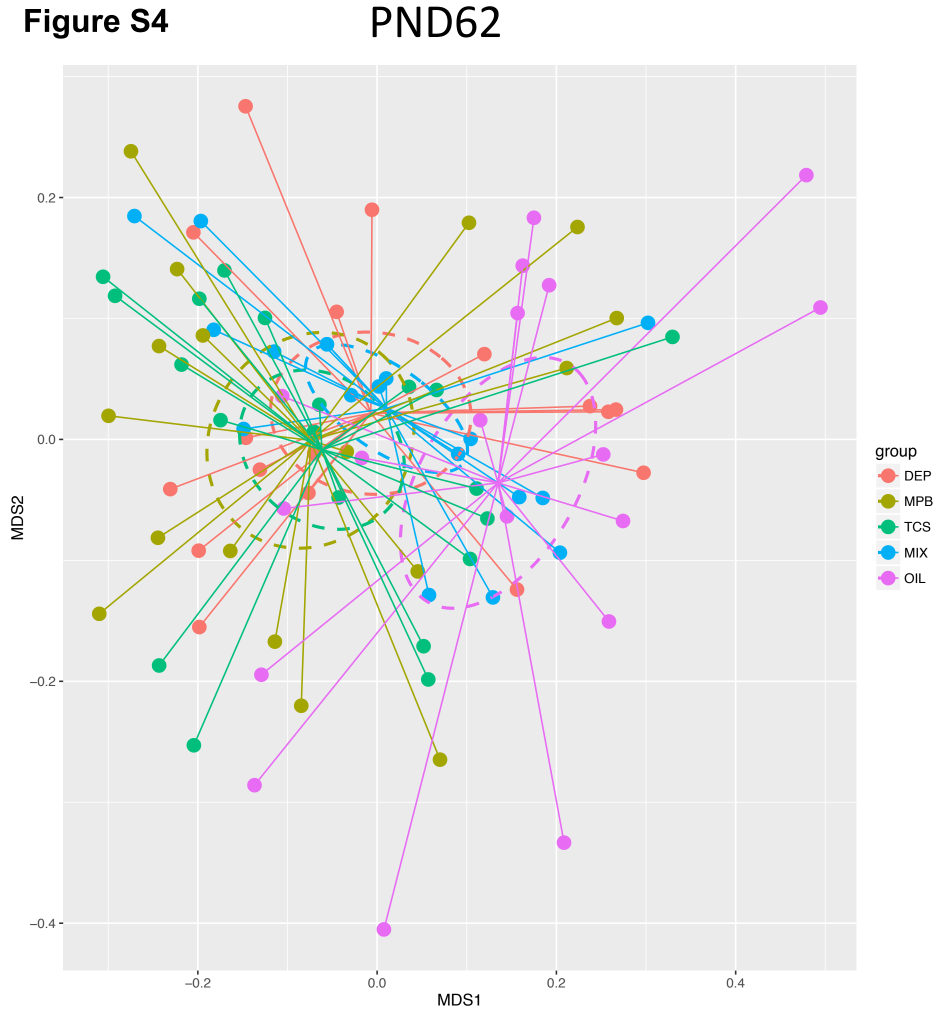
PND62
0.2
0.0
−0.2
−0.4
−0.2
0.0
0.2
0.4
MDS1
MDS2
group
DEP
MPB
TCS
MIX
OIL

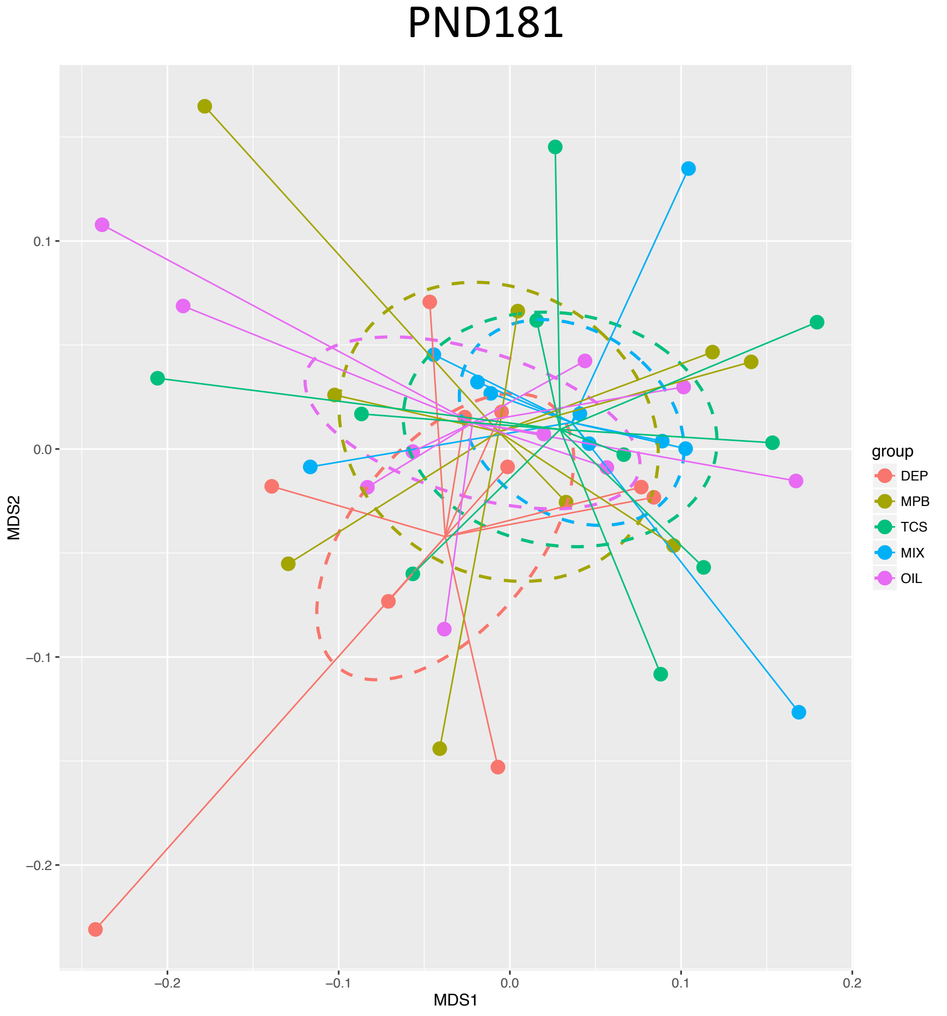
PND181
0.1
0.0
−0.1
−0.2
−0.2
−0.1
0.0
0.1
0.2
MDS1
MDS2
group
DEP
MPB
TCS
MIX
OIL

Figure S5

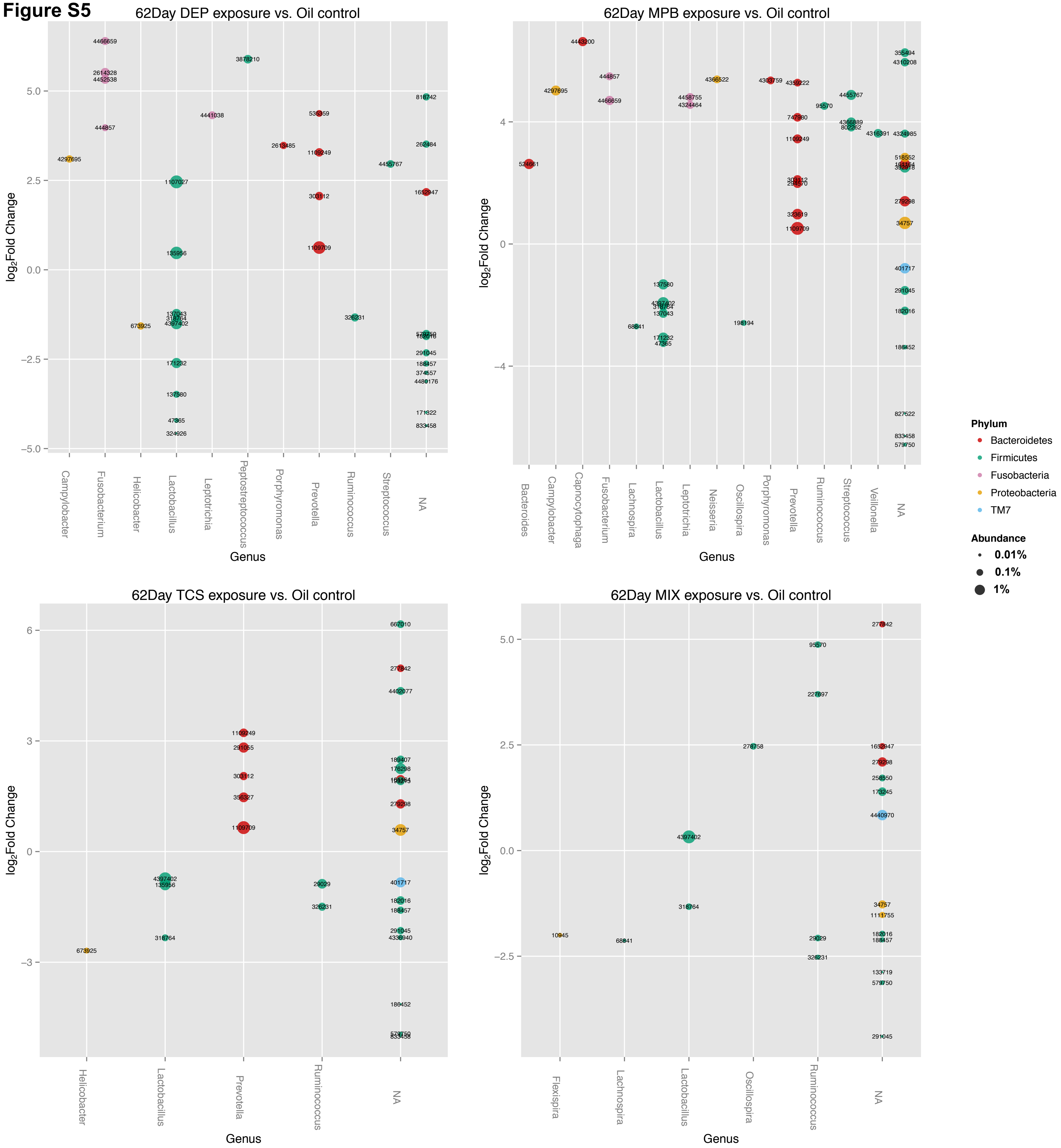

62Day DEP exposure vs. Oil control
62Day MPB exposure vs. Oil control
62Day TCS exposure vs. Oil control
62Day MIX exposure vs. Oil control
log2Fold Change
Genus
Phylum
Bacteroidetes
Firmicutes
Fusobacteria
Proteobacteria
TM7
Abundance
0.01%
0.1%
1%
